# Gamma and Psychological Resilience: Where to Now?

**DOI:** 10.3390/brainsci15090957

**Published:** 2025-09-02

**Authors:** Damian L. Rocks, Christopher F. Sharpley, Vicki Bitsika, Kirstan A. Vessey, G. Lorenzo Odierna, Christopher B. Watson

**Affiliations:** Brain-Behaviour Research Group, University of New England, Armidale, NSW 2351, Australia; drocks@myune.edu.au (D.L.R.); csharpl3@une.edu.au (C.F.S.); kvessey@une.edu.au (K.A.V.); godierna@une.edu.au (G.L.O.); cwatso29@myune.edu.au (C.B.W.)

**Keywords:** psychological resilience, EEG, stress, depression

## Abstract

Because of their potential to enhance pathways for diagnosis and treatment, a great deal of research has been conducted to identify brain biomarkers of mental illnesses such as depression and anxiety. Similarly, the investigation of the biomarkers of those protective factors that help individuals resist, or recover from, these mental illnesses is also directly relevant to clinical practice. One such protective factor is Psychological Resilience (PR) but relatively little is known about its neurobiological underpinnings. A literature search was conducted of electroencephalographic data and PR, revealing seven studies that reported brain electrical activity categorised into bands of specified frequencies. Several studies reported significant associations between PR and alpha band activity, and somewhat less on beta band activity, principally via their roles in emotion regulation and problem solving. However, despite having a major role in many aspects of cognition and brain function, and being implicated in depression, only two studies examined gamma band activity specifically, and their results were equivocal. Several possible reasons for these apparently null results of the association between gamma band activity and PR are discussed, leading to a brief description of gamma, and suggestions for future research into its association with PR.

## 1. Introduction

Although a great deal of research has focused on the neurobiological correlates of depression, anxiety, and other neuropsychiatric disorders [1,2,3,4,5], relatively little attention has been given to the neurobiological correlates of ‘protective’ factors that help individuals avoid or recover from the kind of chronic or acute stress that is often a precursor to these disorders [6,7,8]. Referred to as ‘coping skills’, these protective factors include (among others) optimism, resilience, sense of purpose, mental toughness, and cognitive-behavioural self-help strategies for assisting individuals to recover from the effects of chronic or acute stress and develop long-term attitudes and strategies for succeeding in their life goals. Of these, ‘resilience’, sometimes referred to as ‘psychological resilience’ (PR), has been demonstrated as a major positive influence on mental health following either chronic or acute stress, possibly attributable to its emphasis on thinking patterns and beliefs [9,10,11].

PR has been variously defined as an adaptive response to stressful stimuli [12], a set of coping skills [13], behaviours which buffer deleterious consequences following exposure to adversity [14], or an attitudinal approach inversely related to depression [15]. As such, PR may reflect an organism’s immediate response to stressors [16], the longer-term adaptive psychobiological processes which follow exposure to adversity [17], or to allostatic capacities which protect against the onset of negative sequelae [18].

## 2. Measurement of PR

Most often, PR is measured using self-reporting psychometric tools such as the Connor-Davidson Resilience Scale CDRISC [13]. Defining resilience as a measure of stress-coping ability, the CDRISC provides a means of assessing an individual’s capacity to employ coping strategies [19] or engage with techniques for stress minimization in the face of adversity [20]. Regarded as one of the most reliable measures of assessing resilience characteristics based on a recent meta-analysis of 27 studies [21], the CDRISC is composed of questions designed to gauge different aspects of resilience, with respondents asked to rate their response to 25 items along a 5-point Likert scale (0 to 4), based on feelings experienced over the past month. A score between 0 and 100 is then calculated, with higher scores indicative of greater resilience. These items may then be further delineated into five Resiliency Factors (RF) [15,21] to aid investigative analysis.

PR has been explored across a variety of domains and disciplines [13,22,23] and, while its associations with a range of personality traits are relatively well-established [24], the neurobiological underpinnings of PR are not [16,25]. To date, most empirical studies have focussed on the behavioural and psychosocial characteristics of resilience and stress adaptation [26]. Numerous laboratory-based and/or pre-clinical investigations have identified elements of the psychobiological pathways involved in PR [16,27]. These tend to rely, however, on animal models examining stress responses and fear physiology, with uncertain translation to human populations [28]. That is, although animal studies may inform on the biological mechanisms involved with stress reactivity, PR in humans is multifaceted and involves higher cognitive functions such as purpose, meaning-making, and self-confidence, which add complexity to the nature of what is being examined while also reducing the relevance of cross-species investigations [29,30]. Therefore, developing more human-relevant neurobiological models able to reveal the diverse mechanisms involved in PR holds potential clinical and research significance.

## 3. Reviews of the Neurobiology of PR

There have been several valuable reviews of the neurobiology of resilience, and some of those reviews have focused upon the use of neuroimaging procedures that are relevant to this discussion, albeit with some major limitations. For example, Van der Werff et al. [31] identified 12 studies published prior to 2013 that had focused on resilience in PTSD patients, mostly without adequate healthy control groups. Major findings were that, in comparison to participants who had experienced traumatic stress but had not developed any disorder, participants who qualified for the diagnosis of PTSD exhibited decreased emotional regulation, principally due to less control exerted by the ventral medial prefrontal cortex, hippocampus, and other regions that comprise the “emotion regulation brain circuitries” [31] (p. 10) upon amygdala activity. Although interesting results from structural, resting-state and task-related neuroimaging data were reported, the review authors commented that “neuroimaging research of resiliency is still in its infancy” (p. 1) at that time. A decade later, Tai et al. [32] reviewed 19 fMRI studies of PR that were published prior to 2022, finding that regions of the emotional network (orbitofrontal cortex, anterior cingulate cortex, insula, amygdala) were often reported as having associations with PR, confirming the earlier results published by van der Werff et al. [31] that emotional regulation was the key neurological pathway via which resilience was activated in persons who experienced trauma but did not proceed to develop depression, PTSD, anxiety, and other major disorders. No common brain regions were identified as being linked with PR in the studies reviewed by Tai et al. [32], but other reviews have similarly reported a direct association between emotional regulation and PR [33,34,35].

These reviews were focused upon the associations between PR and brain region structure and activity as detected via blood flow with MRI and fMRI. Other researchers have used fMRI to focus on the connections between a specific network (Salience) or brain region (left orbitofrontal cortex) and PR [36,37]. A similar process that measures the brain’s magnetic fields (Magnetoencephalography, MEG) to detect electrical activity has also been used to detect the correlation between PR and cross-network electrical connectivity between the default mode network and the salience network [14]. These latter types of studies represent a move in methodology from brain region structure and function to measuring the electrical activity of the brain’s ‘wave’ activity at selected frequencies. These frequencies are defined by the ranges of their speed (Hz), and are most commonly collected using electroencephalographic (EEG) procedures which measure the post-synaptic potentials of pyramidal neuron apical dendrites [38] at specific scalp sites. These waves are generally classified according to their associations with specific cognitive states (see Table 1). So that the current state of understanding of the association between PR (as a cognitive state) and brain wave activity (i.e., according to the frequency bands shown in Table 1) could be evaluated, a brief narrative review of the literature was conducted.

## 4. EEG and PR

To ascertain the current state of research on the association between PR and EEG-measured brain waves, a search of the literature between 2000 and 2025 was conducted independently by two authors (DLR, CFS). This 25-year period was chosen because it represented a reasonably long enough duration to encompass major recent research, and because title searches of the years prior to 2000 did not reveal any studies relevant to this review. In fact, only one study was found prior to 2019, with the remaining six studies all being published within the 2019–2025 period (2 × 2019, 1 × 2023, 2 × 2024, 1 × 2025). Studies were selected if they focussed on resilience as a topic of enquiry, used a standardised scale for measuring PR, and reported EEG data on any of the waves listed in Table 1. Seven relevant studies were found, and their details were tabulated independently by the same two authors (DLR, CFS): see Table 2. Because of the limited number of studies identified, a correlational, narrative synthesis was undertaken.

Presented chronologically in Table 2, it is clear that there has been little attention paid to this issue until the last five years. Of the seven studies, five used standardized scales to measure PR and two used standardized scales to measure correlates or derivatives of PR. That is, Curtis et al. [40] drew their sample from children who had/had not been maltreated, and examined the association between positive emotionality and increased emotional regulation, resilient functioning, and frontal region EEG asymmetry. Results indicated that resilient children showed greater alpha activity in their left hemisphere than non-resilient children. The second study that used a non-standardized scale to estimate resilience [41] used the residuals of a regression analysis of scores on the Perceived Stress Scale and the Social Readjustment Rating Scale. Resting EEG data were collected from eight electrodes which measured delta, theta, alpha, and beta wave activity. Only limited verification of the worth of using EEG data in this setting was found—a trend towards significance (*p* = 0.08) in the theta band at electrode P4.

The remaining five studies all applied a standardized scale for measuring PR, four of which used the 25-item Connor Davidson Resilience Scale (CDRISC), and one used the Brief Resilience Scale (6 items). Lee et al. [42] examined alpha, beta, and gamma wave coherence (connectivity) between brain regions in internet gamblers compared to healthy non-gamblers. EEG coherence has been used to detect a range of neurological disorders [43], including autism [44], ADHD [45], anxiety and depression [46], OCD [47], and bipolar disorder [48]. Lee et al.’s [42] results indicated that there was a significant relationship between CDRISC score and alpha coherence in the right hemisphere, but no significant relationships between CDRISC and either beta or gamma coherence.

Paban et al. [49] used the 10-item CDRISC to measure PR in 45 healthy participants and tested for correlations with brain network flexibility in delta, theta, alpha, and beta wave frequencies. Network models describe the brain as a set of ‘nodes’ which represent specific regions in the brain, and a set of ‘edges’, or the connections between these nodes. Paban et al. [49] defined network flexibility as how often a particular node changed its relationships over a given period, thus capturing the dynamics of brain functional networks. Results indicated a negative correlation between PR and brain network flexibility for delta, alpha, and beta bands in specific brain regions, suggesting that the brain’s core networks are less flexible in people with high PR. Paban et al. [49] interpreted this finding as indicative of greater robustness of brain networks in resilient individuals, i.e., their networks were less vulnerable to the adverse effects of stress.

Sharpley et al. [50] used the 25-item CDRISC to classify their 100 community participants, but examined PR at the level of the five factors reported for the CDRISC [13]. Results indicated that different EEG sites were associated with each of the five CDRISC factors, suggesting that different aspects of PR are initiated within different brain regions. That suggestion was confirmed by source location data derived from eLORETA [51], including positive correlations between frontal right alpha power and CDRISC Factor 1 (Personal competence) and CDRISC Factor 2 (Trust in one’s instincts), variability in alpha power across right and left frontal regions in relation to CDRISC Factor 4 (Control), and inverse associations between alpha power and CDRISC Factor 5 (Spiritual influences) across both frontal hemispheres. These findings also argue against the use of the CDRISC as a homogeneous measure of PR and suggest that PR itself may be a multifaceted construct.

Using the same sample as Sharpley et al. [50], Evans et al. [15] also examined the five CDRISC factors, but tested their association with functional connectivity in the alpha and beta bands across the salience network, the default mode network, and the central executive network by reference to eLORETA-derived measures of coherence and phase synchronization. Evans et al. [15] reported that each CDRISC factor exhibited distinct functional connectivity patterns across the default mode network, the central executive network, and the salience network, but with differences between depressed and non-depressed participants, suggesting that depression itself may modulate how resilience is expressed in neural networks.

**Table 2:** Chronological Summary of Reports Regarding Neuroelectrophysiological Correlates of Resilience.

Author, Year, Reference	PR Measure	N, Sample, Age	EEG Data Collected	Summary Outcomes
Curtis et al., 2007 [40]	Combination of depression and internalizing/externalizing behaviour	37 resilient (16 males), *M* age = 10.3 yr. 50 non-resilient (27 males), *M* age = 10.4 yr	Resting state alpha asymmetry	Greater left central hemisphere alpha activity in resilient vs. non-resilient groups.
Lee et al., 2019 [42]	CDRISC	36 healthy males, *M* age = 25.2 yr. 35 Internet Gambling males, *M* age = 23.7 yr	Resting state EEG, alpha, beta, gamma (30–40 Hz) bands	Internet Gambling Ss with low PR had sig. higher alpha coherence in right hemisphere
Paban et al., 2019 [49]	10-item CDRISC	45 (23 males) healthy volunteers =. *M* age = 34.7 yr	Resting state EEG, delta, theta, alpha, beta	Sig. inverse r between PR and network flexibility across cortical regions in the delta, alpha and beta bands.
Sharpley et al., 2023 [50]	CDRISC	100 (54 males) healthy volunteers. *M* age = 32.5 yr	Resting state EEG, frontal alpha asymmetry	Variation in r between EEG frontal alpha and five PR factors across brain regions
Evans et al., 2024 [15]	CDRISC	100 (54 males) healthy volunteers. *M* age = 32.5 yr	Resting state EEG, alpha and beta connectivity across networks	Variation in connectivity r between PR and networks according to PR factor
KeunhoYoo et al., 2024 [41]	Relationship between perceived stress and impact of stress	55 (29 males) healthy volunteers. *M* age = 23.3 yr.	Resting state EEG, delta, theta, alpha beta bands	No sig. correlations between PR and EEG data.
Gupta & Reddy 2025 [52]	Brief Resilience Scale (6 items)	12 (3 males) volunteers. Age range = 18–24 yr	Resting state EEG, delta, theta, alpha, beta, gamma (31–49.5 Hz) bands	Sig. differences between high vs. low PR subgroups for alpha (frontal, right side) and beta (posterior, left sided) power.

Note. CDRISC = Connor-Davidson Resilience Scale; PR = Psychological Resilience.

In the second part of a two-part study, Gupta and Reddy [52] investigated the association between PR (measured via a brief 6-item PR scale) and resting EEG data in the delta, theta, alpha, beta and gamma bands, under eyes open and eyes closed conditions. Spectral analysis showed that the high resilience group also had significantly higher alpha (10 Hz to 12 Hz) activity in the right central, right, and left parietal regions, and significantly higher beta differences in the left central, left parietal, and posterior midline regions. Gupta and Reddy [52] concluded that their results indicated that PR was a composite of the effects of emotional regulation as well as problem solving. Those authors argued that greater beta-wave activity in the prefrontal cortex (PFC), posterior midline region, and left parietal region in high resilience individuals than low resilience individuals implied the former’s greater use of logical judgement of the relative importance of a stressor on the basis of previous history, thus enhancing more effective problem solving. Similarly, higher beta wave activity in the PFC is associated with reduced amygdala activity, thereby contributing to emotional regulation. Gupta and Reddy thus argued that both of these functions were necessary to recover from major stress and avoid mental illness.

## 5. Summary

These seven studies provide some support for the presence of significant relationships between PR and various wavelengths of EEG data collected. Five studies reported significant associations between alpha band activity and (some form of) PR, but for different aspects of alpha activity (Curtis et al., 2007; Lee et al., 2019; Paban et al., 2019; Sharpley et al., 2023; Evans et al., 2024, and Gupta et al., 2025) [15,40,42,49,50,52]. There was also support for the role of beta wave activity in PR (Paban et al., 2019; Gupta et al., 2025) [49,52]. Paban et al. [49] discussed their results in terms of the resilient individual’s ability to bring problem solving and emotional moderation to respond to stressors, and this interpretation of results is congruent with the other studies reviewed in Table 2 that reported significant associations between PR and aspects of alpha and beta. The lack of any significant association between the measure of resilience and EEG data reported by Keunho Yoo et al. [41], compared to some significant findings reported in those studies that used a standardised scale for measuring PR (i.e., the CDRISC), suggests that an important methodological aspect of any future research should be consistent use of such a standardised scale of PR, although the measure used by Curtis et al. [40] was able to produce evidence of a significant association between alpha activity and resilience.

## 6. The Argument for Gamma

While a number of studies in Table 2 reported an association between alpha or beta wave brain activity and PR on the basis of existing evidence that alpha is involved in emotional regulation and beta is related to problem solving, little attention has been given to gamma band activity, with only two studies reporting the collection of gamma wave data (Lee et al., 2019; Gupta et al., 2025) [42,52]. Given the fact that the definitions of gamma in the two studies were not identical (Gupta et al. [52]: 31–49.5 Hz; Lee et al. [42]: 30–40 Hz), and they did not examine the whole range of gamma (i.e., 30–200 Hz), it is difficult to conclusively determine what role gamma might have in PR on the basis of the literature to date, despite the plausible argument that gamma might be associated with PR as it is with other higher-level cognitive processes (see Section 7.2 below). Given the pivotal role that PR plays in terms of mental health beliefs [9,10,11], plus the influence of gamma in cognition [53], it is important to establish whether gamma-wave activity is associated with PR. However, as indicated by Fitzgerald and Watson [54], there has been little consensus about the definition and associations of gamma. Therefore, prior to recommending the experimental investigation of the association between gamma and PR, it is important to define gamma, describe its properties, and explore the hypothetical basis of associations between gamma and PR.

## 7. Gamma Wave Definition and Characteristics

### 7.1. Definition

Gamma oscillations are rhythmic, electrophysiological events that typically fall within the 30–200 Hz frequency range [55], and emanate from the interaction of both excitatory and inhibitory neurons, reflecting ionic changes within perisomatic and synaptic zones [56,57]. Gamma rhythms have transient characteristics [58] which can appear across multiple brain regions in response to inter-neuronal signaling [59]. In addition, gamma oscillations can emerge via long-range (inter-areal) synchronization of neural networks, which may provide a framework for system-based communication and coordinated neuronal activity [56].

Although other oscillatory bands such as alpha and beta appear as constant waves during specific cognitive states (see Table 1), gamma oscillations are typically produced in sudden bursts [56] from neuronal clusters which may function as “circuit generators” [55] (p. 2), displaying at times random, short-lived characteristics [56] which serve to link both local and distant neuronal populations. Periodic bursting of gamma oscillations appears to reflect the systematic firing of adjacent neural circuits [55,60,61] but can also be indicative of broader brain regional activities underlying augmentation or synchronization of cognitive processes and synaptic signaling [62]. Intercellular (local) as well as system-wide activity patterns that drive gamma waves are possibly involved in connecting brain regions [63], enabling coordinated neuronal spike firing and entrainment, and inhibitory/excitatory cycling, which may help parse sensory inputs [64] and modulate attention [62,65]. It is reasonable to hypothesize that these characteristics of gamma might contribute to an association with the problem solving and emotion modulation aspects of PR.

### 7.2. Gamma Associations

Unlike other oscillatory bands, gamma rhythms display uniquely stochastic properties which may correlate with a range of cognitive events [56]. For example, gamma oscillations are known to emerge in vegetative states [66], during heightened arousal or cognitive processing [58,64], or during episodes of neural synchrony [29]. Although previous data demonstrate the importance of gamma oscillations in terms of brain activity [67,68], a diversity of opinion exists as to their psychological [54,66], operational [55] and even physiological significance [58,65].

Basar [66] argued that, rather than being indicative of any single cognitive process, gamma oscillations may provide a substrate upon which is built a “universal code” [66] (p. 104) facilitating cerebral communication. This is an important perspective which may help link gamma oscillatory activity to psychological states such as consciousness and attentional selection [69], or the emotion regulation and problem solving aspects of PR. The possible role of gamma as a modulator of disparate brain functions is supported by evidence suggesting that gamma oscillations may function as both critical “building blocks” [66] (p. 102) which link neural systems and networks [63], as well as neuromodulatory agents with causal [55,58,62] and homeostatic influence [70,71].

The role of gamma in neural function is also supported by a significant correlation between gamma-wave dysfunction and disorders involving either cognitive impairment and/or sensory processing [59,66,72]. In addition, gamma-wave stimulation can have a therapeutic impact across a variety of brain disorders [59,73]. Furthermore, recent evidence has led some researchers to propose that, rather than being mere signatures of neural interactions, gamma oscillations also perform a neuromodulatory function across brain networks [55,57,58,62], thus contributing to psychological health through the combined interaction of exogenous inputs (socialization/affiliation/conscious experience) as well as circuit-based homeostatic operations (perfusion/neurochemical expression/glial function). This bidirectional—and potentially causal—effect resembles the role of PR when the individual is under stress, and argues for exploration of the role of gamma oscillations in the expression of PR.

## 8. Gamma and PR: Where to from Here?

These insights have critical relevance for the study of PR. As Connor and Davidson [13] recognized, healthy neural function is fundamental for resilience. If gamma cycles modulate homeostasis, then their (dys)function must impact coping skills and resiliency. Thus, an understanding of how gamma interacts with PR has the potential to contribute to therapeutic models. That is, as described in Section 1, PR is established as a ‘buffer’ against the development of anxiety, depression, and PTSD following chronic stress, and so the possible link between gamma activity and PR suggests that therapy procedures aimed at regularising gamma may boost PR. For example, as reviewed by Pascual-Leone and Bartres-Faz [74], brain-site specific neurofeedback or non-invasive brain stimulation might be employed with the individual who is suffering chronic stress and the early signs of mental illness. These treatments may be offered in clinical [75] and non-clinical settings [76] due to the robustness of their methodology.

It has been established that a significant gap exists between what is known about PR and how gamma activity relates to either PR measures or associated therapeutic protocols. Whether gamma oscillations are modified by PR (or vice versa) is unknown, but the relationship between PR and gamma remains a vital target for future research. For example, changes in gamma band expression may denote a current (or impending) alteration in an individual’s tendency to engage in prosocial behaviours, which may then impact their PR. Examination of gamma power spectral densities for their correlation with PR, which might then be matched to relevant brain sites, could assist in understanding the potential dynamics involved. Comparing these PR-gamma correlations within narrow-band gamma frequencies (30–50 Hz, 50–70 Hz, etc., to 200 Hz.) might also help ‘map’ specific gamma characteristics against PR, a process that could then be extended to the subfactors of PR as well as total CDRISC score. Application of more precise source of localization technique (e.g., eLORETA) to determine whether any specific neural structures or brain sites may emit/suppress gamma oscillations, or are correlated with PR and its components, would expand understanding of the neurobiological substrates of PR.

As a caveat, due to the PR measures used in five of the seven studies reported in Table 2, the CDRISC became the instrument by which PR was defined in this review, but there are many other similar scales that might be used, perhaps delivering different associations with various EEG-based wave bands. Finally, as was argued in Section 1, PR is one of several coping skills, but one that has a wealth of research supporting its ‘buffer’ role, and so a focus on it is justifiable for the purpose of this review. However, because several coping skills are strongly correlated with each other (e.g., mental toughness and PR: *r* = 0.59 [77]), there is an argument to examine the neural correlates of (a) the common content of these coping skills, and (b) the independent content of those coping skills. Because, for example, a correlation coefficient of 0.59 means that one of the two measures accounts for just under 36% of the variance in the other, it might be expected that there would be an overlap within the brain-based EEG data for each scale also. However, as an alternative argument, nearly two-thirds of the variance in each of these two constructs is not common, and so their investigation separately might reveal unique associations between aspects of each and brain wave activity.

## 9. Conclusions

Psychological resilience has been described as a function of bio-psychosocial balance. If gamma oscillations are the building blocks of cognitive function, and diminished gamma-wave activity is strongly correlated with cognitive impairment and psychological disorder, then resilience as a feature of mental health/balance might be measured through fluctuations in gamma oscillations. Optimal gamma-wave expression may be a requisite for PR to emerge, and the efficacy of psychological interventions may be measured against changes in gamma-band activity. Further studies linking the potential modulatory effects of gamma oscillations with mental health are required, with the aim of creating a more robust understanding of the neurobiological underpinnings of PR, as well as the relationship between gamma oscillations and overall wellbeing.

## Figures and Tables

**Table 1:** Brain wave frequencies and cognitive states in adults [39].

Name	Frequency (Hz)	Cognitive State
Delta	0–4	Deepest relaxation, restorative, dreamless sleep
Theta	4–8	Deep relaxation, deep meditation
Alpha	8–12	Calm wakefulness, resting state eyes closed, meditation
Beta	12–30	Alert, active thinking, focus, attention, anxiety
Gamma	30–200	Heightened perception, learning, information processing functions.

## Data Availability

All the data reviewed here are available in the original publications.
